# *Theileria luwenshuni* and Novel *Babesia* spp. Infections in Humans, Yunnan Province, China

**DOI:** 10.3201/eid3109.241919

**Published:** 2025-09

**Authors:** Rong Xiang, Chun-Hong Du, Yi-Lin Zhao, Zhi Luo, Miao Li, Dan-Ni Zeng, Fan Wang, Chao-Bo Du, Yi Sun, Qiao-Cheng Chang, Jia-Fu Jiang

**Affiliations:** Author affiliations: State Key Laboratory of Pathogen and Biosecurity, Academy of Military Medical Sciences, Beijing, China (R. Xiang, Y.-L. Zhao, D.-N. Zeng, Y. Sun, J.-F. Jiang); Yunnan Institute of Endemic Disease Control and Prevention, Dali, China (C.-H. Du, Z. Luo, M. Li, F. Wang, C.-B. Du ); School of Public Health at Shantou University, Shantou, China (Q.-C. Chang)

**Keywords:** piroplasmosis, Theileria luwenshuni, Babesia spp., parasites, vector-borne infections, zoonoses, Yunnan, China

## Abstract

Piroplasmid parasites such as *Theileria luwenshuni* protozoa pose a global threat to both animal and human health. However, human theileriosis remains underexplored compared to infections caused by *Plasmodium* and *Babesia* species parasites. We investigated potential hemoparasite infections among 1,721 persons with fever, anemia, or both in Yunnan Province, China. Molecular detection identified 13 cases positive for *T. luwenshuni* protozoa, of which 5 patients were further confirmed by Western blot antibody analysis. We also identified 6 babesiosis cases, 3 infections with *B. microti* and 3 with novel *Babesia* spp. Subsequent vector and host investigations in the vicinity of the index cases revealed *T. luwenshuni* protozoa in 1 tick and 53 livestock animals. Of note, 3.3% combined vector-host samples tested positive for genetically diverse *Babesia* species. Our findings highlight the endemic circulation of *T. luwenshuni* and *Babesia* spp. parasites in southwest China, underscoring their importance as emerging public health concerns.

Piroplasmorida, a group of tickborne hemoparasites within the phylum Apicomplexa, includes diverse protozoa species such as *Babesia* spp. and *Theileria* spp., which are responsible for causing babesiosis and theileriosis in humans and animals (*1*,*2*). Human babesiosis is a globally recognized parasitic zoonosis that primarily targets red blood cells. Parasite transmission to humans occurs predominantly through the bite of an infected tick; however, alternative transmission routes include blood transfusion, perinatal transmission, and organ transplantation (*3*). Since 1957, the number of human babesiosis cases has increased, posing a growing global public health challenge (*4*). In China, ≈317 cases of human babesiosis or asymptomatic infections had been reported by 2022 (*5*). That relatively low number is likely because of underdiagnosis and limited clinical recognition of these protozoan infections. Rare human infections with hemogregarines of the genus *Hepatozoon*, traditionally considered animal pathogens, have been also detected in immunocompromised patients in Russia, suggesting potential zoonotic spillover of diverse apicomplexan parasites (*6*).

*Theileria* spp. parasites primarily infect ruminants such as cattle and sheep, as well as various wild animals (*7*). Historically, *Theileria* spp. have not been considered pathogenic to humans. However, a 2014 serosurvey in Italy reported IgG reactivity against *T. equi* antigens in veterinary practitioners, indicating potential human exposure, particularly among persons at higher risk for infection (*8*,*9*). That finding has garnered attention because zoonotic *Theileria* species were widespread in livestock.

Yunnan Province, located in southwestern China, provides an ideal habitat for various tick species and their host animals because of its distinct geographic features, dense vegetation, and high biodiversity. Those characteristics make the region a hotspot for tickborne diseases (*10*,*11*). In Tengchong, along the China–Myanmar border, 8 cases of human babesiosis caused by *Babesia microti* and 2 case co-infections with *B. microti* and *Plasmodium* parasites were confirmed in 2013 (*12*). To enhance understanding of the prevalence of tickborne protozoa in China, we studied patients experiencing fever and anemia in Yunnan Province and traced potential sources of infection by examining protozoan prevalence in domestic animals, small wild animals, and ticks within the affected areas. Our goal was to further characterize the epidemiologic and clinical features of piroplasmid parasite infections and identified their possible sources.

## Materials and Methods

### Identification of Piroplasmosis in Patients

We conducted a retrospective investigation among participants experiencing unexplained fever or anemia across 10 counties in Yunnan Province, China, during May 2017–June 2020. Demographic data, medical history, and epidemiologic exposure history had been collected through a structured questionnaire. We retrieved data on clinical manifestations, underlying medical conditions, laboratory test results, treatments, and outcomes from medical records; 2 investigators cross-validated the data. The patients’ blood samples were collected at various time points during their hospital stay; a portion of each patient’s blood samples were immediately processed for blood smear preparation, and the residual blood was stored at −80°C until nucleic acid extraction for batch PCR amplification and downstream analysis. The Ethics Committee of the Yunnan Provincial Institute of Endemic Disease Control approved the study (approval no. 2016-005), which was conducted in accordance with medical research regulations in China. All participants provided written informed consent before their inclusion in the study.

### Source Tracing Investigation

As part of the study, we conducted retrospective testing on ticks, livestock, and small mammals from areas surrounding the participants. Wild small mammals were captured using snap traps, and aseptic tissue samples, including liver and spleen, were collected and stored at −80°C for subsequent analysis. We taxonomically identified the captured animals to the species level on the basis of external morphology, coloration, measurements, and dental characteristics. We collected whole-blood samples from livestock via jugular vein puncture using EDTA anticoagulant tubes and stored them at −20°C until DNA extraction. We manually removed ticks from livestock and collected host-seeking ticks by flag-sweeping vegetation at the same sampling sites. An entomologist identified tick species. We preserved tick samples at −80°C before DNA extraction.

### PCR Amplification and Sequencing

We extracted DNA from human blood, livestock blood, small mammal tissues, and tick samples using the DNeasy Blood & Tissue Kit (QIAGEN, https://www.qiagen.com) in accordance with manufacturer’s instructions. To detect *Theileria* and *Babesia* parasites, we performed nested PCR targeting the 18S rRNA gene using outer primers Piro0F/Piro6R and inner primers Piro1F/Piro5.5R (*13*,*14*), followed by agarose gel electrophoresis and Sanger sequencing. We amplified additional genetic loci, including the 5.8S rRNA (303 bp), internal transcribed spacer region (1,300 bp), P32 immunodominant protein gene (875 bp), and cytochrome oxidase subunit I (1,200 bp). We conducted concurrent testing for other potential infections with *Rickettsia* spp., *Borrelia burgdorferi*, *B. recurrentis*, and *Bartonella* spp., which could potentially be transmitted by similar transmission routes or cause similar symptoms, to exclude differential diagnoses (Appendix 1 Table 1).

### Phylogenetic Analysis

We performed phylogenetic analysis using sequences assembled with the CLC Main Workbench version5.5 (QIAGEN). We conducted comparative analysis against sequences in GenBank using BLAST (https://blast.ncbi.nlm.nih.gov). We conducted phylogenetic analysis of all sequences using MEGA version 11.0 software (https://www.megasoftware.net). We constructed phylogenetic trees using the neighbor-joining method with the p-distance model based on 1,000 bootstrap replicates. 

### Morphologic and Serologic Testing

We stained thin peripheral blood smears collected from the participants with Giemsa and examined under a light microscope (Olympus, https://evidentscientific.com). We used the recombinant *T. uilenbergi* immunodominant protein (rTuIP) as the diagnostic antigen for Western blot analysis (*15*,*16*). Anhui Global Gene Technology Company (Chuzhou, China) conducted protein expression and purification using the pQE31 vector as the expression vector and BL21 (DE3) as the expression host. Tengchong People’s Hospital (Tengchong County, China) provided serum samples collected for rTuIP antibody detection from some patients with suspected protozoan infections and used serum samples from healthy persons as negative controls.

We separated recombinantly expressed TuIP protein (20 μg) using 12% sodium dodecyl sulfate-polyacrylamide gel electrophoresis and transferred it onto a polyvinylidene difluoride membrane (0.45 μm pore size). To block nonspecific binding, we incubated the membrane with 5% skimmed milk powder in Tris-buffered saline containing Tween 20 for 2 hours. We used patient serum samples diluted at various ratios (1:25–1:800) as primary antibodies; serum samples from healthy persons diluted at 1:100 served as negative controls. We then incubated the membrane overnight at 4°C. We applied horseradish peroxidase–labeled human IgG secondary antibody, diluted at 1:5,000, for a 1-hour incubation at room temperature. After washes in Tris-buffered saline containing Tween 20, we detected the signal using an enhanced chemiluminescence developing reagent.

## Results

### Identification of Piroplasmosis in Participants

**Figure 1:**
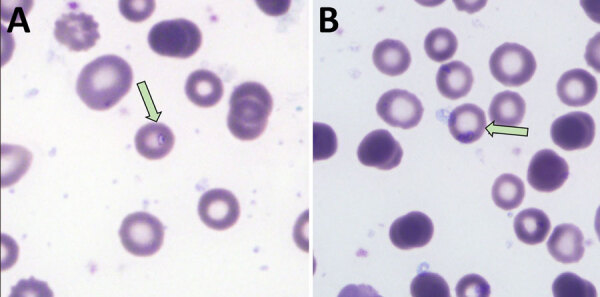
Photomicrographs from peripheral blood smears of a patient (patient 2) who tested positive for *Theileria luwenshuni* parasite in study of piroplasmorida in humans, southwest China, May 2017–June 2020. Arrows indicate typical ring forms; single (A) and multiple (B) parasitism was observed.

We screened a total of 1,721 participants from 10 counties in Yunnan Province for protozoan infections; 1,362 participants were experiencing fever, and 359 had anemia, (Appendix 1 Table 2). Among those, we identified 18 inpatients and 1 outpatient to have suspected protozoan infections by 18S rRNA sequencing of blood samples; amplification for additional *T. luwenshuni* genetic loci was not successful. Meanwhile, molecular testing for *Rickettsia* spp., *B. burgdorferi* sensu lato, *B. recurrentis*, and *Bartonella* spp. yielded uniformly negative results. We examined peripheral blood smears from the 19 suspected cases for intraerythrocytic parasites using Giemsa staining; patient 2 tested positive for piroplasmosis parasites (Figure 1). No parasites were detected in the blood smears of the remaining patients.

**Figure 2:**
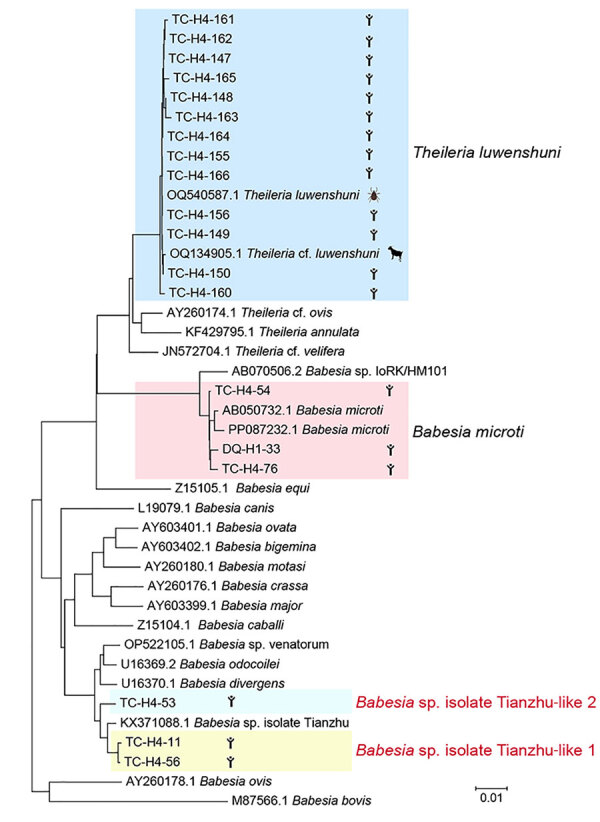
Phylogenetic analysis of 18s rRNA (1,600-bp) gene sequences of *Theileria* and *Babesia* spp. isolates in study of piroplasmorida in humans, southwest China, May 2017–June 2020. Colored shading represents groups of isolates by strain. Scale bar represents 0.01 substitutions per site.

We successfully amplified and sequenced the 18S rRNA gene of *T. luwenshuni* parasites from 13 blood samples, yielding sequences ≈1,600 bp long. Those sequences exhibited a similarity range of 99.28%–100% among the samples; we deposited them into GenBank (accession nos. PQ720759–71). Similarity analysis indicated that the *T. luwenshuni* samples shared 99.69% identity with a reference sequence isolated from a *Haemaphysalis longicornis* tick (GenBank accession no. OQ540587.1) and 98.60% identity with a *T. luwenshuni* isolate from goats (GenBank accession no. OQ134905.1), both collected in Shandong Province, China. Phylogenetic analysis further showed that the sequences clustered within the same monophyletic branch (Figure 2; Appendix 1 Table 3).

We successfully amplified and sequenced the 18S rRNA gene of *Babesia* spp. from 6 blood samples, yielding sequences ≈1,600 bp long. We submitted those sequences to GenBank (accession nos. PQ720772–5, PQ722837, PQ722838). Phylogenetic analysis demonstrated that samples DQ-H1-33, TC-H4-76, and TC-H4-54 clustered with multiple *B. microti* strains from reference sequences (GenBank accession nos. KF410825.1 and AB050732.1), exhibiting a high genetic similarity (Figure 2). In addition, samples TC-H4-11 and TC-H4-56 clustered with *Babesia* Tianzhu–like subgroups that were identified in white yaks in China (*14*). Meanwhile, sample TC-H4-53 clustered with another subgroup, which suggested that TC-H4-53 may represent an uncharacterized or novel *Babesia* variant or strain (Figure 2).

**Figure 3:**
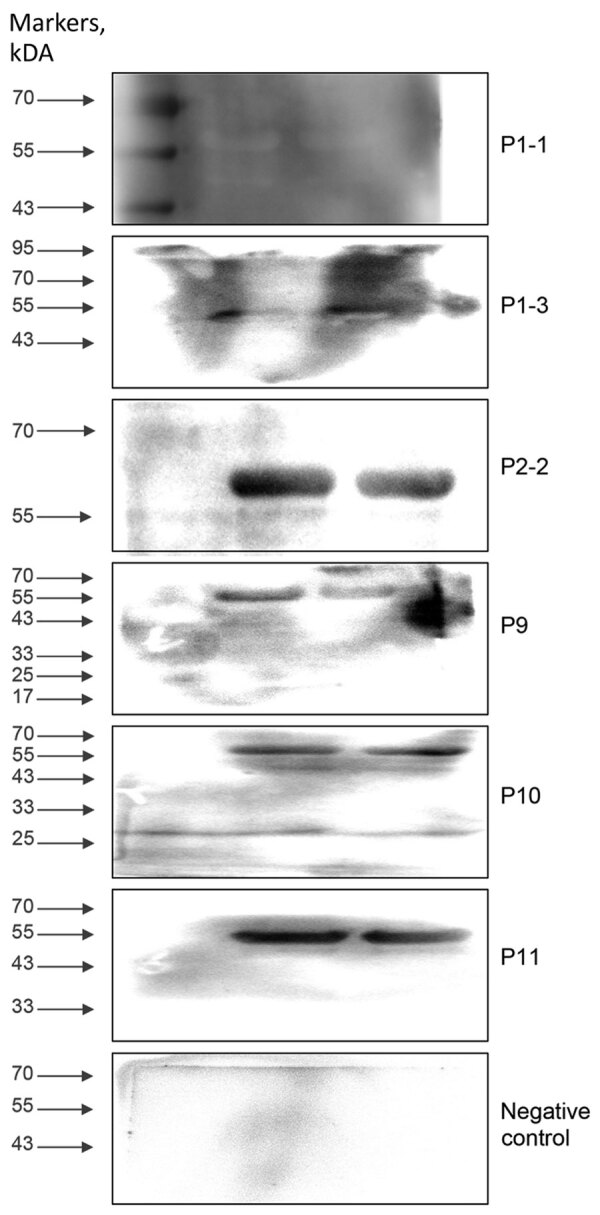
Western blot analysis of the target protein (55-kDa) expression in the serum samples from 5 patients with suspected protozoan infection in study of piroplasmorida in humans, southwest China, May 2017–June 2020.

Western blot analysis confirmed the diagnostic utility of the rTuIP protein for detecting *T. luwenshuni* infections. Of 11 serum samples from participants with suspected hemoprotozoan infections, 6 samples (from 5 patients) exhibited characteristic rTuIP-specific bands at a 1:25 dilution (Figure 3). Of note, longitudinal samples from patient 1 (P1-1 and P1-3) both exhibited positive reactivity. Control samples from healthy participants showed no target protein–specific bands.

Analysis of longitudinal antibody responses also revealed dynamic seroconversion patterns over time (Appendix 2 Figure, panel A). Sample P1-1 exhibited faint reactivity at 1:25 dilution but was negative at >1:50. In contrast, subsequent samples from the same patient (P1-2 and P1-3) displayed strong immunoreactivity across all consecutive doubling tested dilutions (1:50–1:800) (Appendix 2 Figure, panel A), indicating the maturation of antibody titers. We saw similar consistently high-titer responses in patient 2 at 2 timepoints, with clear bands visible from 1:50–1:800 dilutions (Appendix 2 Figure, panel B).

### Epidemiologic and Clinical Characteristics of the Patients

Among the 13 patients with suspected *T. luwenshuni* infection (Appendix 1 Table 4), the median age was 40 (interquartile range [IQR] 32.5–68.0) years; 10 (77%) patients were male and 13 (23%) female. The median hospital stay was 13 (IQR 7.5–17.0) days. All patients were engaged in farming. Underlying conditions noted at admission included renal failure in 5 (38%) patients, upper gastrointestinal bleeding in 5 (38%) patients, and a history of cancer in 3 (23%) patients; 1 patient reported prior blood transfusion history. The primary clinical manifestations were anorexia (8 [62%] patients), listlessness (8 [62%] patients), malaise (5 [38%] patients), and melena (4 [31%] patients). Other reported symptoms included vomiting (4 [31%] patients), dizziness (3 [23%] patients), and palpitations (3 [23%] patients); cough, jaundice, splenomegaly, and weight loss were each observed in 1 patient (8%). One patient reported neurologic symptoms (Appendix 1 Table 4). Laboratory findings revealed anemia in all 13 patients and leukocytosis in 2 (15%) patients. Elevated serum levels of aspartate aminotransferase, alanine aminotransferase, or gamma-glutamyl transferase were present in 1 patient (8%). All patients received general supportive and symptomatic treatment; 4 (31%) patients underwent blood transfusions, and 5 (38%) patients required hemodialysis (Table 1).

**Table 1:** Characteristics of 18 patients hospitalized for *Theileria luwenshuni* and *Babesia* *spp.* infection, China*

Characteristic	*T. luwenshuni* cases, n = 13	*Babesia* *spp.* cases, n = 5
Age, y (IQR)	40 (32.5–68.0)	56 (25.5–59.5)
Sex		
M	10 (77)	4 (80)
F	3 (23)	1 (20)
Length of hospital stay, d (IQR)	13 (7.5–17.0)	10 (5.5–10.5)
Occupation as farmer	13 (100)	5 (100)
Medical history and underlying disease		
Renal failure	5 (38)	1 (20)
Cancer	3 (23)	0
Upper gastrointestinal bleeding	5 (38)	3 (60)
Clinical manifestations†		
Nonspecific symptoms		
Dizziness	3 (23)	4 (80)
Malaise	5 (38)	4 (80)
Cough	1 (8)	0
Jaundice	1 (8)	0
Splenomegaly	1 (8)	0
Weight loss	1 (8)	0
Cardiovascular manifestations		
Chest discomfort	1 (8)	1 (20)
Palpitations	3 (23)	1 (20)
Dyspnea	2 (15)	1 (20)
Gastrointestinal manifestations		
Anorexia	8 (62)	2 (40)
Vomiting	4 (31)	0
Bloating	1 (8)	1 (20)
Stomachache	1 (8)	0
Hematemesis	3 (23)	0
Melena	4 (31)	3 (60)
Neurologic manifestations		
Syncope	1 (8)	1 (20)
Listlessness	8 (62)	2 (40)
Laboratory findings		
Anemia	13 (100)	5 (100)
Leukocytosis	2 (15)	1 (20)
Elevated serum AST or ALT or γ-GGT concentration	1 (8)	0

*Values are no. (%) patient except as indicated. ALT, alanine aminotransferase; AST, aspartate aminotransferase; γ-GGT, gamma-glutamyl transferase; IQR, interquartile range. †Patients could report >1 symptom.

Among the 5 patients with suspected *Babesia* infections (Appendix 1 Table 5), median age was 56 years (IQR 25.5–59.5 years); 4 (80%) patients were male and 1 (20%) female. Similar to the *T. luwenshuni* cohort, all patients were engaged in farming. Underlying conditions were renal failure in 1 patient (20%) and a history of upper gastrointestinal bleeding in 3 patients (60%). Median hospitalization duration was relatively short at 10 days (IQR 5.5–10.5 days). Two patients (40%) had a history of blood transfusion; 1 (20%) patient had a history of gastric hemorrhage or hypertension. The most frequently reported symptoms were nonspecific. Four (80%) patients experienced dizziness and malaise, 3 (60%) patients melena, and 2 (40%) listlessness. Bloating, chest discomfort, palpitations, and dyspnea were all present in 1 (20%) patient. Of note, none of the patients in this group reported vomiting, jaundice, or splenomegaly. Laboratory tests revealed anemia in all patients and leucopenia in 1 (20%) patient. Elevated serum levels of aspartate aminotransferase, alanine aminotransferase, or gamma-glutamyl transferase were not detected in any case. In terms of treatment, 3 (60%) patients received blood transfusions, and 1 patient (20%) required hemodialysis (Table 1).

### Traceability Survey

**Figure 4:**
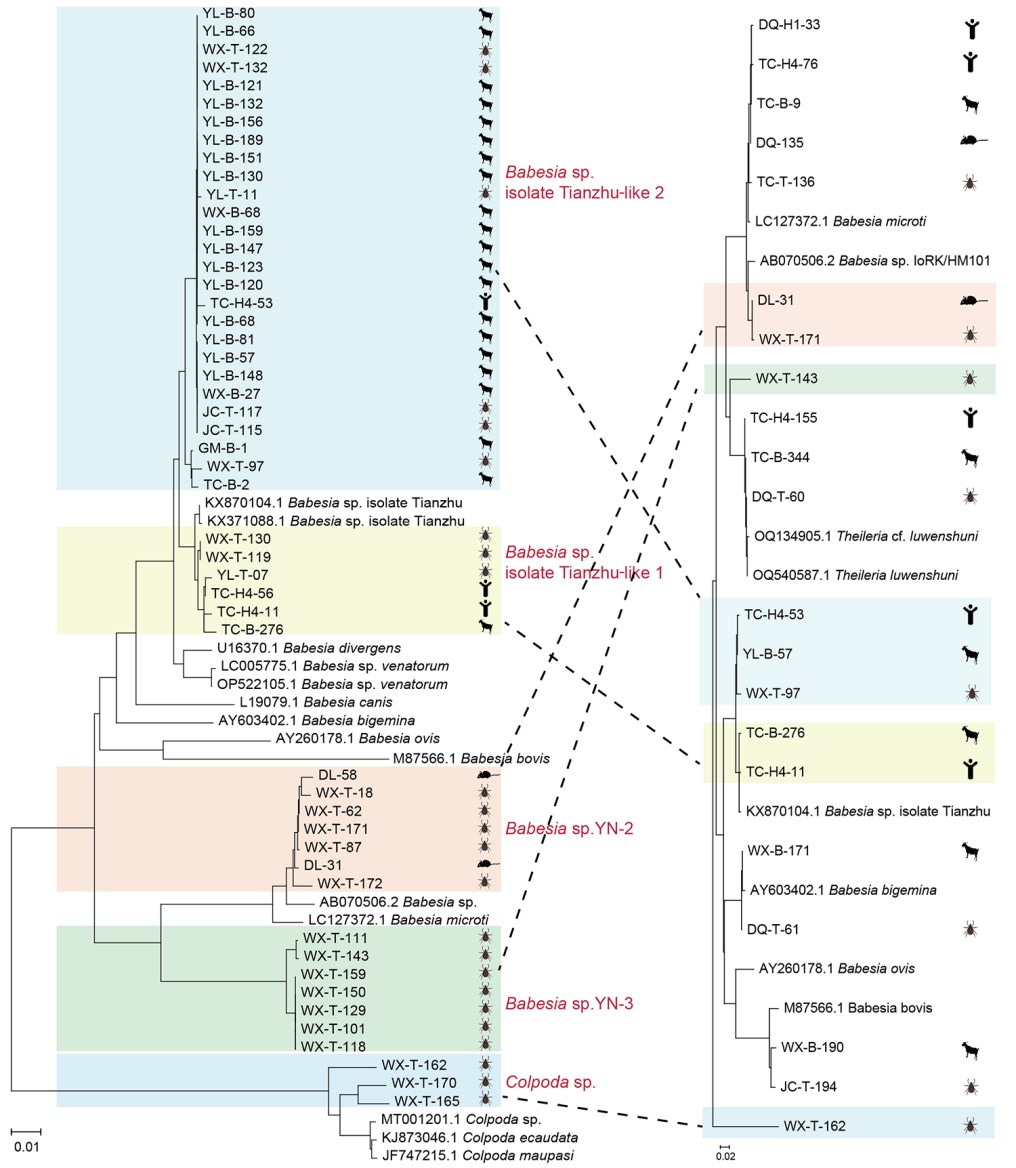
Phylogenetic analysis of 18s rRNA (1,600-bp) gene sequences of *Theileria* and *Babesia* spp. isolates in study of piroplasmorida in humans, southwest China, May 2017–June 2020. Tree on the left highlights the sequences of novel variants/strains (accession nos. PQ722834–6, PQ722839–82). Tree on the right shows classification and distribution of all detected organisms across various hosts and vectors. Colored shading represents groups of isolates by strain. Dotted lines indicate the correspondence between different organisms, illustrating their ecologic and evolutionary connections. Left scale bar represents 0.01 substitutions per site; right scale bar represents 0.02 substitutions per site.

**Figure 5:**
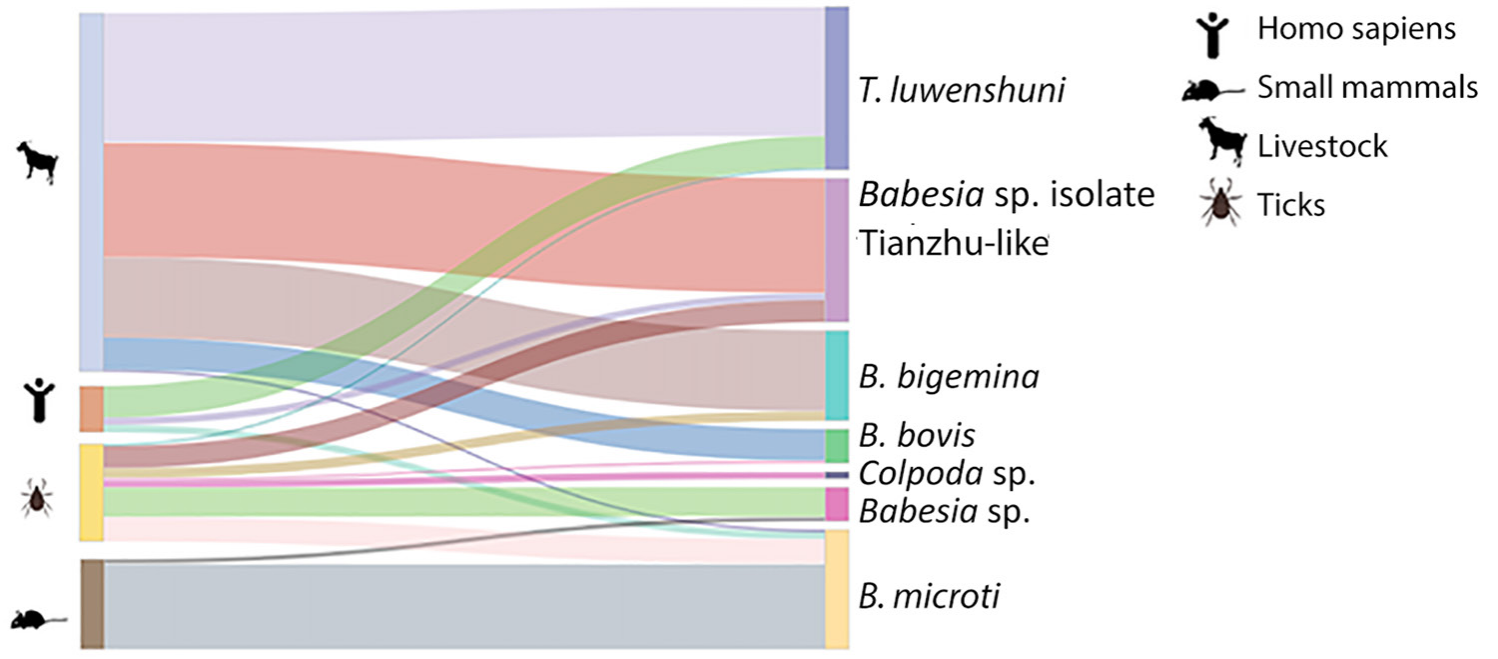
Characterization of host and vector associations determined from phylogenetic analysis of *Theileria* and *Babesia* spp. isolates in study of piroplasmorida in humans, southwest China, May 2017–June 2020.

We performed PCR analyses on 2,100 livestock, 1,530 wild small mammals, and 1,516 ticks collected from 10 counties in Yunnan Province to detect Piroplasmorida infections (Table 2). We used the same protocol previously optimized for human diagnostics. Among the livestock samples, 147 (7.00%) tested positive. We constructed a phylogenetic tree using 18S rRNA sequences (≈1,600 bp) to classify the positive samples into 5 species: *B. microti*, *Babesia* sp. Tianzhu-like, *B. bigemina*, *B. bovis*, and *T. luwenshuni* (Figures 4, 5). We detected 1 strain of *B. microti* and 2 strains of *T. luwenshuni* parasites in livestock from Tengchong County. The overall positivity rate for small mammals was 2.42%. We identified *B. microti* as the predominant species. Phylogenetic analysis identified a variant, tentatively named *Babesia* sp. YN-2, in small mammals (Figure 4). Among tick samples, 37 (2.44%) tested positive for Piroplasmorida; detected species including *B. microti*, *B. bigemina*, *Babesia* sp. Tianzhu-like, *B*. *bovis*, *Babesia* sp., *Colpoda* sp., and *T. luwenshuni*. Phylogenetic analysis further classified the novel *Babesia* sp. into 2 clades: *Babesia* sp. YN-2 and *Babesia* sp. YN-3.

**Table 2:** Piroplasmorida infections by pathogen in humans, livestock, small mammals, and ticks, southwest China, May 2017–June 2020*

Pathogen	No. positive/no. tested										
	Tengchong	Puer	Jianchuan	Yunlong	Dali	Gengma	Fugong	Deqin	Weixi	SL	Total
Human with fever											
* Babesia microti*	0/583	0/171	0/28	0/64	0/141	0/325	0/10	1/40†			1/1,362
Total	0/583	0/171	0/28	0/64	0/141	0/325	0/10	1/40†			1/1,362
Human with anemia											
* B. microti*	2/174	NA	NA	0/21	0/151	0/13	NA	NA	NA	NA	2/359
*B. tianzhu* like	3/174	NA	NA	0/21	0/151	0/13	NA	NA	NA	NA	3/359
* Theileria luwenshuni*	13/174	NA	NA	0/21	0/151	0/13	NA	NA	NA	NA	13/359
Total	18/174	NA	NA	0/21	0/151	0/13	NA	NA	NA	NA	18/359
Livestock											
* B. microti*	1/758	0/10									1/2,100
*B. tianzhu* like	2/758	NA	26/175	16/212	NA	1/54	NA	NA	2/200	0/450	47/2,100
* B. bigemina*	20/758	NA	0/175	0/212	NA	0/54	NA	NA	8/200	5/450	33/2,100
* B. bovis*	7/758	NA	0/175	0/212	NA	0/54	NA	NA	2/200	4/450	13/2,100
* T. luwenshuni*	53/758	NA	0/175	0/212	NA	0/54	NA	NA	0/200	0/450	53/2,100
Total	82/758	0/10	26/175	16/212		1/54	0/139	0/102	12/200	9/450	147/2,100
Small mammals											
* B. microti*	8/299	3/10	0/212	19/177	0/126	NA	0/135	3/346	2/99	0/126	35/1,530
*Babesia* sp.	0/299	0/10	0/212	0/177	2/126	NA	0/135	0/346	0/99	0/126	2/1,530
Total	8/299	3/10	0/212	0/177	0/126	NA	0/135	3/346	2/99	0/126	37/1,530
Ticks											
* B. microti*	8/405	0/10	0/284	2/127	NA	0/187	NA	0/144	0/359	NA	10/1,516
* B. bigemina*	0/405	0/10	3/284	0/127	NA	0/187	NA	1/144	0/359	NA	4/1,516
*B. tianzhu* like	0/405	0/10	2/284	2/127	NA	0/187	NA	0/144	5/359	NA	9/1,516
* B. bovis*	0/405	0/10	1/284	0/127	NA	0/187	NA	0/144	0/359	NA	1/1,516
*Babesia* sp.	0/405	0/10	0/284	0/127	NA	0/187	NA	0/144	12/359	NA	12/1,516
* T. luwenshuni*	0/405	0/10	0/284	0/127	NA	0/187	NA	1/144	0/359	NA	1/1,516
Total	405	10	284	127	NA	0/187	NA	144	359	NA	37/1,516

*NA, not available; SL, Shangri-La. †These patients are from the same autonomous prefecture, and the case records do not specify the county-level units.

Genetic analysis of the *Babesia* sp. Tianzhu-like isolates identified in patients, livestock, and ticks yielded 2 major clades with high homology (96.11%–98.67%) but distinct variations of 21–62 bp. We tentatively designated those clades as *Babesia* sp. isolate Tianzhu-like 1 and *Babesia* sp. isolate Tianzhu-like 2. The phylogenetic tree depicted the relationships among *Babesia* and *Theileria* species collected from various hosts and vectors. Of note, we identified *B. microti* in multiple host and vector types, indicating its high adaptability across diverse environments (Figure 5). In addition, we detected the novel *Babesia* sp. YN-2 in both small mammals and ticks, suggesting the existence of a potential transmission cycle between wildlife and arthropod vectors. The detection of *Babesia* sp. isolate Tianzhu-like parasites across humans, livestock, and ticks highlights its broad host range and geographic distribution. Similarly, the widespread occurrence of *T. luwenshuni* parasites in humans, livestock, and ticks underscores the species’ potential for transmission among multiple hosts.

## Discussion

This study identified 4 tickborne hemoparasites with human infectivity, including a zoonotic infection caused by 2 known pathogenic agents and 2 novel *Babesia* species. It characterized the diversity and complexity of tickborne protozoa in ticks, small wild animal hosts, and livestock in southwestern China. Our findings are critical for public health and the enhancement of parasitic disease surveillance systems in Yunnan Province. The study presented a clinically relevant finding of human *Theileria* infections, corroborated through molecular diagnostics and Western blot serology, which were validated previously (*16*) and were further confirmed by our serologic titers and longitudinal testing, with no cross-reactivity against other tickborne pathogens.

A previous serosurvey in Italy (*8*) reported IgG reactivity against *T. equi* antigens in veterinary practitioners, indicating potential human exposure to this pathogen, particularly among persons at heightened risk for infection. That result raised considerable interest because of the prevalence of zoonotic *Theileria* species in livestock. Of note, our study reported a series of human infections with *T. luwenshuni* parasites, previously recognized as primarily affecting ruminants, with less reported pathogenicity in humans.

Our study also identified potential host-vector transmission routes involving domestic animals and ticks. Genetic analysis indicated that *T. luwenshuni* isolates exhibited high similarity to vector-derived strains from Shandong Province, in the east of China. That finding indicates a widespread distribution, an emerging epidemic trend, and the potential for cross-species transmission of these organisms. Those results provide valuable insights for future epidemiologic investigations targeting affected populations, hosts, and vectors across diverse regions.

In this study, we detected >6 species of Piroplasmorida in livestock, small mammals, and ticks: *B. microti*, *Babesia* sp. Tianzhu-like, *B. bigemina*, *B. bovis*, *Babesia* sp., and *T. luwenshuni*. We observed genetic diversity within *Babesia* sp. Tianzhu-like; homology was 96.11%–98.67% and 21–62 bp variations. Four of those species are known or suspected to exhibit explicit pathogenicity. Of note, we identified *Babesia* sp. Tianzhu-like 1 in patients, livestock, and ticks, with homology of 99.10% –99.61%. However, the exact infection source remains uncertain for some patients who lacked a clear history of tick bites but reported previous blood transfusions. In addition, most patients were immunocompromised, raising questions about the potential for opportunistic infections. Similar to *B. venatorum* and *B. microti* (*17*), asymptomatic carriage of *T. luwenshuni* and other *Babesia* spp. parasites may occur in healthy persons. Furthermore, as the endemic regions expand, *T. luwenshuni* parasites may emerge as a more frequent complication in immunosuppressed hosts, akin to patterns observed in human babesiosis (*18*). Enhanced surveillance, particularly among blood donors in this region, is imperative (*19*).

Since the earliest report of *Theileria* parasites in Sichuan Province in 1958, >4 species have been documented in China: *T. luwenshuni*, *T. unilenbergi*, *T. ovis*, and *T. annulata*. Among them, *T. luwenshuni*, designated in 2007 (*20*,*21*), has been a species recognized for its high pathogenicity in sheep and goats. The presence of *T. luwenshuni* parasites has now been confirmed in multiple provinces, including Yunnan, Hubei, Henan, Gansu, Jilin, Hunan, and Shandong (*20*). Of note, the prevalence of *T. luwenshuni* parasites in goats in Shandong Province has been reported to reach 81.5%, higher than rates previously reported in small ruminants and deer in central and northwestern China (*22*–*25*). Those findings suggest an emerging epidemic trend of *T. luwenshuni* infections in humans across multiple regions.

*B. microti* is the predominant species causing human babesiosis in the United States. In China, it has been associated with >100 reported human infections, most occurring in Guangxi Province (*9*). Of note, 10 *B. microti* infections were identified in a study of 449 patients with fever in Tengchong County, Yunnan Province, along the China–Myanmar border (*12*). *Babesia* sp. isolate Tianzhu was initially discovered in Tianzhu Tibetan Autonomous County, northwestern China, in 2017. In our study, we identified this agent in both ticks and water buffalo, demonstrating a close genetic relationship to isolates obtained from 2 human patients (Figure 2); that finding suggests that *Babesia* sp. isolate Tianzhu may present a public health risk.

A limitation of this study is that *T. luwenshuni* infections were identified in immunocompromised patients, and as a retrospective investigation, it was not possible to establish causal relationships between the protozoan infection and patient symptoms or clinical features. Specifically, whether the observed anemia resulted directly from protozoan-induced red blood cell damage or from comorbid conditions (e.g., bleeding disorders or renal failure) remains unclear. Future research should prioritize prospective cohort studies at in sentinel hospitals involving similar patients. Such studies should exclude anemia caused by renal failure or other underlying conditions using interventions such as erythropoietin combined with iron therapy. Those patients should receive antiprotozoal therapy with monitoring of parameters, such as reticulocyte counts and L-lactic dehydrogenase levels, to assess treatment efficacy.

In conclusion, we used molecular biology techniques, including PCR, serology, and phylogenetic analysis, to characterize the diversity and potential risks of tickborne protozoa in Yunnan Province, China. Our findings confirmed the endemic circulation of *T. luwenshuni* and multiple *Babesia* spp. parasites in southwestern China. Further investigation of *T. luwenshuni* infection will elucidate transmission dynamics, clinical impact, and targeted prevention strategies, as well as its implications for public health. Clinicians in this region should remain aware of these emerging public health concerns.

Appendix 1 Additional data about *Theileria* and *Babesia* spp. isolates in study of piroplasmorida in humans, southwest China, May 2017–June 2020.

Appendix 2 Additional information about *Theileria* and *Babesia* spp. isolates in study of piroplasmorida in humans, southwest China, May 2017–June 2020.

## References

[1] Almazán C, Scimeca RC, Reichard MV, Mosqueda J. Babesiosis and Theileriosis in North America. Pathogens. 2022;11:168. 10.3390/pathogens11020168 35215111 PMC8874406

[2] Karasartova D, Gureser AS, Gokce T, Celebi B, Yapar D, Keskin A, et al. Bacterial and protozoal pathogens found in ticks collected from humans in Corum province of Turkey. PLoS Negl Trop Dis. 2018;12:e0006395. 10.1371/journal.pntd.0006395 29649265 PMC5916866

[3] Schnittger L, Rodriguez AE, Florin-Christensen M, Morrison DA. Babesia: a world emerging. Infect Genet Evol. 2012;12:1788–809. 10.1016/j.meegid.2012.07.004 22871652

[4] Skrabalo Z, Deanovic Z. Piroplasmosis in man; report of a case. Doc Med Geogr Trop. 1957;9:11–6. 13427667

[5] Chen MX, Xue JB, Ai L, Song P, Cai YC, Chen JX. Epidemic status and research progress of babesiosis in China [in Chinese]. J Trop Dis Parasitol. 2022;20:149–57. 10.3969/j.issn.1672-202.2022.03.005

[6] Shuĭkina EE, Beĭer TV, Sergiev VP, Iastrebova RI. [Detection of hemogregarin of the genus *Hepatozoon* in patients in Russia] [in Russian]. Med Parazitol (Mosk). 2004; (4):3–6. 15689126

[7] Sivakumar T, Hayashida K, Sugimoto C, Yokoyama N. Evolution and genetic diversity of *Theileria.* Infect Genet Evol. 2014;27:250–63. 10.1016/j.meegid.2014.07.013 25102031

[8] Gabrielli S, Calderini P, Cassini R, Galuppi R, Tampieri MP, Pietrobelli M, et al. Human exposure to piroplasms in Central and Northern Italy. Vet Ital. 2014;50:41–7. 10.12834/VetIt.1302.13 24715592

[9] Chen Z, Li H, Gao X, Bian A, Yan H, Kong D, et al. Human Babesiosis in China: a systematic review. Parasitol Res. 2019;118:1103–12. 10.1007/s00436-019-06250-9 30770979

[10] Madison-Antenucci S, Kramer LD, Gebhardt LL, Kauffman E. Emerging tick-borne diseases. Clin Microbiol Rev. 2020;33:e00083–18. 10.1128/CMR.00083-18 31896541 PMC6941843

[11] Zhao GP, Wang YX, Fan ZW, Ji Y, Liu MJ, Zhang WH, et al. Mapping ticks and tick-borne pathogens in China. Nat Commun. 2021;12:1075. 10.1038/s41467-021-21375-1 33597544 PMC7889899

[12] Zhou X, Li SG, Chen SB, Wang JZ, Xu B, Zhou HJ, et al. Co-infections with *Babesia microti* and *Plasmodium* parasites along the China-Myanmar border. Infect Dis Poverty. 2013;2:24. 10.1186/2049-9957-2-24 24090043 PMC3819642

[13] Kawabuchi T, Tsuji M, Sado A, Matoba Y, Asakawa M, Ishihara C. *Babesia microti*-like parasites detected in feral raccoons (*Procyon lotor*) captured in Hokkaido, Japan. J Vet Med Sci. 2005;67:825–7. 10.1292/jvms.67.825 16141672

[14] Liu J, Guan G, Li Y, Liu A, Luo J, Yin H. A molecular survey of *Babesia* species and detection of a new *Babesia* species by DNA related to *B. venatorum* from white yaks in Tianzhu, China. Front Microbiol. 2017;8:419. 10.3389/fmicb.2017.00419 28352260 PMC5349112

[15] Liu Z, Li Y, Salih DE, Luo J, Ahmed JS, Seitzer U, et al. Validation of a recombinant protein indirect ELISA for the detection of specific antibodies against *Theileria uilenbergi* and *Theileria luwenshuni* in small ruminants. Vet Parasitol. 2014;204:139–45. 10.1016/j.vetpar.2014.05.010 24912957

[16] Liu Z, Wang Z, Yin H, Luo J, Zhang B, Kullmann B, et al. Identification of *Theileria uilenbergi* immunodominant protein for development of an indirect ELISA for diagnosis of ovine theileriosis. Int J Parasitol. 2010;40:591–8. 10.1016/j.ijpara.2009.10.011 19900458

[17] Ruebush TK II, Juranek DD, Chisholm ES, Snow PC, Healy GR, Sulzer AJ. Human babesiosis on Nantucket Island. Evidence for self-limited and subclinical infections. N Engl J Med. 1977;297:825–7. 10.1056/NEJM197710132971511 561308

[18] Heller HM. Babesiosis in immunosuppressed hosts: pathogenesis, diagnosis and management. Curr Opin Infect Dis. 2024;37:327–32. 10.1097/QCO.0000000000001038 39109671

[19] Jiang JF, Zheng YC, Jiang RR, Li H, Huo QB, Jiang BG, et al. Epidemiological, clinical, and laboratory characteristics of 48 cases of “*Babesia venatorum*” infection in China: a descriptive study. Lancet Infect Dis. 2015;15:196–203. 10.1016/S1473-3099(14)71046-1 25539588

[20] Yin H, Liu Z, Guan G, Liu A, Ma M, Ren Q, et al. Detection and differentiation of *Theileria luwenshuni* and *T. uilenbergi* infection in small ruminants by PCR. Transbound Emerg Dis. 2008;55:233–7. 10.1111/j.1865-1682.2008.01031.x 18666967

[21] Yin H, Schnittger L, Luo J, Seitzer U, Ahmed JS. Ovine theileriosis in China: a new look at an old story. Parasitol Res. 2007;101(Suppl 2):S191–5. 10.1007/s00436-007-0689-2 17823827

[22] Wang BH, Du LF, Zhang MZ, Xia LY, Li C, Lin ZT, et al. Genomic characterization of *Theileria luwenshuni* strain Cheeloo. Microbiol Spectr. 2023;11:e0030123. 10.1128/spectrum.00301-23 37260375 PMC10434005

[23] Li Y, Chen Z, Liu Z, Liu J, Yang J, Li Q, et al. Molecular identification of *Theileria* parasites of northwestern Chinese Cervidae. Parasit Vectors. 2014;7:225. 10.1186/1756-3305-7-225 24885179 PMC4029935

[24] Li Y, Zhang X, Liu Z, Chen Z, Yang J, He H, et al. An epidemiological survey of *Theileria* infections in small ruminants in central China. Vet Parasitol. 2014;200:198–202. 10.1016/j.vetpar.2013.07.023 24365241

[25] Zhang X, Liu Z, Yang J, Chen Z, Guan G, Ren Q, et al. Multiplex PCR for diagnosis of *Theileria uilenbergi, Theileria luwenshuni*, and *Theileria ovis* in small ruminants. Parasitol Res. 2014;113:527–31. 10.1007/s00436-013-3684-9 24241125

