# Supplementary material for: Theileria luwenshuni and Novel Babesia spp. Infections in Humans, Yunnan Province, China
Source: Emerg Infect Dis. 2025 Sep;31(9):1764–73. doi: 10.3201/eid3109.241919 (PMC12407207; doi:10.3201/eid3109.241919)
Supplement: Appendix 2 — Additional information about Theileria and Babesia spp. isolates in study of piroplasmorida in humans, southwest China, May 2017–June 2020. [file 24-1919-Techapp-s2.pdf]

Article DOI: https://doi.org/10.3201/eid3109.241919

EID cannot ensure accessibility for supplementary materials supplied by authors. Readers who have difficulty accessing supplementary content should contact the authors for assistance.

Theileria luwenshuni and Novel *Babesia* spp. Infections in Humans, Yunnan Province, China

Appendix 2

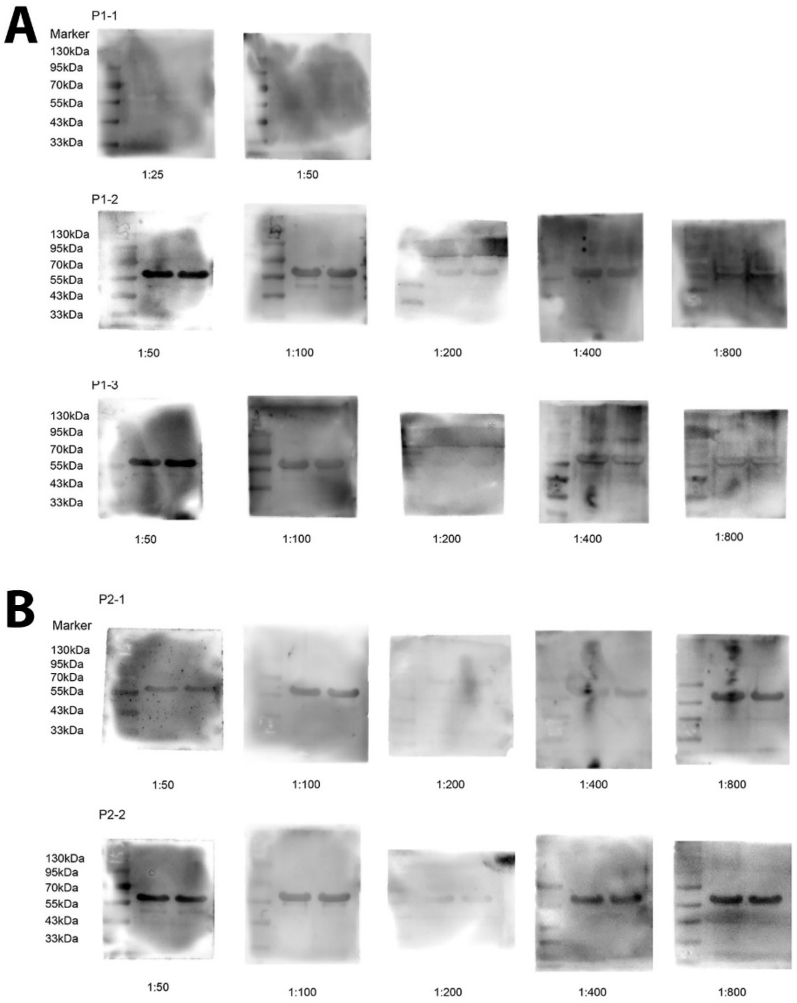

Appendix Figure. Western blot analysis of target protein in study of piroplasmorida in humans, southwest China, May 2017–June 2020. A) Western blot analysis of the target protein (55 kDa)

expression in serum samples from patient 1 across 3 consecutive time points. B) Western blot analysis of the target protein (55 kDa) expression in the serum from patient 2 across 3 consecutive time points.
